# Dose-Dependent Effects of Melatonin on the Viability, Proliferation, and Differentiation of Dental Pulp Stem Cells (DPSCs)

**DOI:** 10.3390/jpm12101620

**Published:** 2022-10-01

**Authors:** Shankargouda Patil, Ahmed Alamoudi, Bassam Zidane, Khalid J. Alzahrani, Fuad M. Alzahrani, Hamsa Jameel Banjer, Rodolfo Reda, Thodur Madapusi Balaji, Shilpa Bhandi, A. Thirumal Raj, Luca Testarelli

**Affiliations:** 1 Department of Maxillofacial Surgery and Diagnostic Science, Division of Oral Pathology, College of Dentistry, Jazan University, Jazan 45142, Saudi Arabia; 2 Centre of Molecular Medicine and Diagnostics (COMManD), Saveetha Dental College and Hospitals, Saveetha Institute of Medical and Technical Sciences, Saveetha University, Chennai 600077, India; 3 College of Dental Medicine, Roseman University of Health Sciences, South Jordan, UT 84095, USA; 4 Oral Biology Department, Faculty of Dentistry, King Abdulaziz University, Jeddah 21589, Saudi Arabia; 5 Department of Restorative Dentistry, Faculty of Dentistry, King Abdulaziz University, Jeddah 21589, Saudi Arabia; 6 Department of Clinical Laboratories Sciences, College of Applied Medical Sciences, Taif University, Taif 21944, Saudi Arabia; 7 Department of Oral and Maxillo-Facial Sciences, Sapienza University of Rome, 00161 Rome, Italy; 8 Department of Dentistry, Tagore Dental College and Hospital, Chennai 600127, India; 9 Department of Restorative Dental Sciences, Division of Operative Dentistry, College of Dentistry, Jazan University, Jazan 45142, Saudi Arabia; 10 Department of Cariology, Saveetha Dental College and Hospitals, Saveetha Institute of Medical and Technical Sciences, Saveetha University, Chennai 600077, India; 11 Department of Oral Pathology and Microbiology, Sri Venkateswara Dental College and Hospital, Chennai 600130, India

**Keywords:** dental pulp stem cells, melatonin, mesenchymal stem cells

## Abstract

(1) Background: Dental pulp stem cells (DPSCs) are derived from pulp tissue lodged within human teeth and are mesenchymal in origin. These DPSCs have been demonstrated to dissociate into clusters of various cell lineages and are very easy to isolate, culture, and expand. Melatonin, a multifaceted molecule with a spectrum of effects in the human body, is known to influence stem cell viability, proliferation, and differentiation, but little is known about the impact melatonin has on the capacity of DPSCs to differentiate into adipocytes, osteocytes, and chondrocytes. The primary objective of this research was to explore the impact that melatonin has on proliferation, and the capacity of DPSCs to differentiate into adipocytes, osteocytes, and chondrocytes. (2) Methodology: DPSCs were extracted from 12 healthy human teeth, cultured, and expanded. Flow cytometry was performed to examine the surface stem cell markers. Further, melatonin was added to the cultured DPSCs in various concentrations, to assess cytotoxicity using an MTT assay. Following this, the DPSCs were tested for their proliferative ability, as well as adipogenic, osteogenic, and chondrogenic differentiation capabilities under the influence of variable concentrations of melatonin. (3) Results: DPSCs obtained from human teeth demonstrated surface characteristics of mesenchymal stem cells, as shown by the positive expression of CD105, CD90, and CD73 markers. An MTT cytotoxicity assay revealed that melatonin was well tolerated by the cells at low (1 µM) and high (25 µM) concentrations. Assessment of DPSC cell differentiation elucidated that melatonin at 1 µM and 25 µM concentrations with the induction media stimulated DPSCs to differentiate into osteocytes, but did not have much influence on adipogenic and chondrogenic differentiation. (4) Conclusions: Melatonin could be used in stem cell and tissue engineering applications for osteogenic differentiation of DPSCs and could protect these cells due to its cytoprotective, immunomodulatory, and antioxidant roles, in addition to being an osteopromoter molecule.

## 1. Introduction

Stem cell (SC) research has advanced significantly in recent years. It is now clinically feasible to collect stem cells from an individual, increase those cells, and differentiate them into the necessary phenotype for use during a regenerative surgery. The science of tissue engineering uses stem cells, scaffolds, and growth factors to create tissues ex vivo for transplantation into a living patient [1]. The oral cavity continues to be a readily available source of stem cells that may be easily harvested from a person. The dental pulp [2], gingiva [3], periodontal ligament [4], and deciduous teeth [5] are recognized as major stem cell niches in the human body and as sources of stem cells. SCs that are isolated from pulp tissue are referred to as DPSCs and exhibit a variety of mesenchymal SC-specific markers, including CD105, CD90, and CD73 [6]. They are bereft of SC markers (hematopoietic) and express true characteristics of mesenchymal stem cells. In addition, they are able to develop into a variety of cell types, including hepatocytes, neurons, osteoblasts, and chondrocytes, and have a high degree of viability and proliferation capacity [7,8]. Furthermore, it is interesting that DPSCs interact favorably with a variety of scaffolds and induction factors employed in regenerative medicine [9].

Melatonin has been studied for its effects on stem cells in relation to growth factors and stimulatory chemicals in the field of stem cell biology [10]. Melatonin, having the chemical formula “N-acetyl-5-hydroxytryptamine”, is primarily a product of pinealocytes, the chief cells residing in the pineal gland [11]. The pineal gland produces melatonin during the dark phases of the day via a series of enzymes that mediate its synthesis from tryptophan and serotonin. It is now understood that melatonin is produced by a number of human body tissues, including the gastrointestinal tract [12], testicles [13], retina [14], salivary glands [15], and gingiva [16]. Melatonin is thought to be a crucial regulator of circadian rhythm [17] and also involved in bodily processes, including anti-oxidant defense [18], immune modulation [19], cancer prevention [20], and osteoprotection [21]. Therefore, melatonin may be considered to be one of the essential molecules of the human system.

Studies on melatonin’s effects on stem cells have been conducted in the past, and it has been discovered that melatonin promoted the capacity of different stem cell types to proliferate [10]. Cancer stem cells have been shown to be significantly affected by melatonin [22]. Melatonin has been shown to promote osteogenic differentiation and proliferation in DPSCs [23]. There has been some evidence that melatonin could stimulate DPSCs to differentiate into hepatocytes and neurons [24]. Melatonin impacts differentiation in addition to protecting stem cells from oxidative stress and acting as a cytoprotectant [25,26,27]. However, no study has attempted an in-depth investigation on the impact of different melatonin concentrations on the proliferative effect as well as the DPSCs’ ability to differentiate among different types of cells. Hence, in the present study, we aimed to thoroughly isolate and characterize DPSCs from human teeth, and to evaluate melatonin cytotoxic effects on cultured DPSCs. Further, we also determined how different melatonin concentrations impacted the ability of DPSCs to proliferate and differentiate into osteogenic, chondrogenic, and adipogenic tissues.

## 2. Materials and Methods

The current study received permission from the Institutional Review Board of the College of Dentistry at Jazan University (CODJU-19215, 6 August 2020). After describing the scope of the study, patients were recruited who were undergoing outpatient maxillofacial surgery and diagnostic sciences. Prior to enrolment, the patients provided written, informed consent. According to the study design, individuals between the ages of 14 and 25 who needed third molar extractions for orthodontic purposes, but were otherwise in good general and periodontal health, were included in the study. The exclusion criteria included patients with poor periodontal health, multiple dental lesions, or a history of periodontal therapy, antibiotics, and analgesics intake in the last 6 months. Women who were either pregnant or lactating were also excluded.

### 2.1. Sample Collection

As mentioned earlier, patients requiring third molar extractions for orthodontic purposes were enrolled in the present study (*n* = 12, 6 males and 6 females). The extraction procedure was performed under local anesthesia, the pulp was separated from the teeth following access opening under sterile aseptic conditions, and for further processing, the sample was transferred directly to the molecular biology laboratory.

### 2.2. Culture and Proliferation of Human Dental Pulp Stem Cells

Separating the dental pulp stem cells from the pulp tissue samples (*n* = 12) and characterization for MSC characteristics was conducted using the explant culture system based on a previous narration [28]. Briefly, pulp tissue was cut into small pieces using a blade, positioned in 25 mm polystyrene cell culture plates, and covered completely using enough volume of fetal bovine serum (FBS) (Gibco, Baltimore, MD, USA). The explant specimen consisted of FBS incubated for 24 h at 30 °C under 5% CO_2_, and this was followed by maintaining the entire dental pulp stem cells (DPSCs) culture complex in Dulbecco’s modified Eagle medium (Invitrogen, Carlsbad, CA, USA), to which 20% FBS with antifungal-antibacterial medication was added under similar atmospheric conditions. This was restocked two times in seven days with regular monitoring of the growth, health, and phenotype of the cells via an inverted phase-contrast microscope. Once the cells exhibited from 70 to 80% confluence, they were detached using 0.25% trypsin and ethylenediaminetetraacetic acid solution (Invitrogen, Carlsbad, CA, USA) and transferred into a bigger 25 cm^2^ culture vessel (Nunc, Rochester, NY, USA). Then, DPSCs were detached with trypsin solution followed by continuous passaging for growth, thereby, the passage 4 cells were considered for further experimentation.

### 2.3. Flow Cytometry-Based Surface Marker Characterization of DPSCs

The cell surface markers were examined using a previously reported protocol [28]. Confluent DPSCs were extracted using trypsin, rinsed twice with phosphate-buffered saline (PBS), and then incubated for 30 min at 4 °C with anti-human CD73-APC, anti-human CD90-APC, anti-human CD105-APC, anti-human CD34-PE, anti-human CD45-FITC, and anti-human human leukocyte antigen-DR isotype-APC antibodies (BergischGladbach, Germany). Using phosphate-buffered saline, antibody-marked cells were washed twice, and ten thousand cells per specimen or batch were collected using the flow cytometer “Attune NxT” (Thermo Fisher Scientific, Waltham, MA, USA). Following that, isotype controls were taken into account for the detection and distinction of +ve and -ve indications.

### 2.4. 3-(4,5-Dimethylthiazol-2-yl)-2,5-diphenyltetrazolium Bromide (MTT) Test of Dental Pulp Stem Cells Subsequent Melatonin Treatment

To assess melatonin’s cytotoxicity on DPSCs at various concentrations (0.5 µM, 1 µM, 2.5 µM, 5 µM, 10 µM, 25 µM, 50 µM, and 100 µM), the DPSCs were plated in a cell culture plate with 96 wells (1 × 10^4^ cells per well) and subjected to melatonin treatment at three different treatment durations, i.e., 24, 48, and 72 h. After that, each well was added with the MTT (Sigma-Aldrich Corporation, St. Louis, MO, USA) before being incubated for 4 h. Then, the media were removed, and each well received 100 µL of dimethyl sulfoxide (Sigma-Aldrich Corporation, St. Louis, MO, USA). Then, a spectrophotometer was used to measure the optical density at 570 nanometers (CA, USA).

### 2.5. Growth Curve Plotting

To verify the differentiation capacity of the DPSCs, 1 × 10^4^ cells were plated into a 12-well cell culture plate and treated with two specific concentrations of melatonin (1 µM and 25 µM). The number of cells was assessed on alternate days for a total duration of 13 days using an inverted microscope [24,28] followed by plotting the growth curve by considering cell numbers for 13 days.

### 2.6. Adipogenic Differentiation of Dental Pulp Stem Cells

DPSCs were seeded at a density of 2500 cells/cm^2^ into a 24-well cell culture plate and treated with adipocyte-inducing medium (Dulbecco’s modified Eagle medium containing ten percent fetal bovine serum, 10 micromoles insulin, 1 µM dexamethasone, 0.5 millimoles 3-isobutyl-1-methylxanthine, and 200 micromoles indomethacin) (Sigma-Aldrich Corporation, St. Louis, MO, USA). At every twenty-four hours, the medium was replaced twice every seven days until 21 days [28]. Adipogenic differentiation was performed by fixation of mature adipose cells using 4% paraformaldehyde and further adding 0.3% oil red O for one hour. Four experimental groups were created to assess adipogenic differentiation: Group 1, control; Group 2, induction media; Group 3, 1 µM of melatonin in induction media; and Group 4, 25 µM of melatonin in induction media.

### 2.7. Osteogenic Differentiation of Dental Pulp Stem Cells

The DPSCs (2500 cells/cm^2^) were seeded into a 24-well cell culture plate (NY, USA) with osteogenic differentiation media, comprised of Dulbecco’s modified Eagle medium with 0.1 micromoles of dexamethasone, 10 millimoles of beta-glycerolphosphate, 50 micromoles of ascorbic acid 2-phosphate, and 1% antibiotic-antimycotic (Sigma-Aldrich Corporation, St. Louis, MO, USA). Regular replacement of the osteocyte-inducing solution was carried out twice every seven days. Subsequently, 21 days later, to analyze the osteogenic lineage differentiation and mineralization assessment, the cells were fixed and the von Kossa histological staining method AgNO_3_ was applied [28]. Four experimental groups were created to assess osteogenic differentiation: Group 1, control; Group 2, induction media; Group 3, 1 µM of melatonin in induction media; and Group 4, 25 µM of melatonin in induction media.

### 2.8. Chondrogenic Differentiation of Dental Pulp Stem Cells

The DPSCs (2500 cells/cm^2^) were added to a 24-well cell culture plate (NY, USA) enriched with a medium inducing the synthesis of chondrocytes. The medium was comprised of Dulbecco’s modified Eagle medium with 100 nM of dexamethasone, 1X-ITS, fifty micrograms per milliliters of ascorbic acid 2-phosphate, one millimole of sodium pyruvate, 10 nanograms per milliliters of TGF-β3, and 40 micrograms per milliliters of L-proline (Sigma-Aldrich Corporation, St. Louis, MO, USA). These cultures were incubated for a duration of one month at 37 °C and maintained in a 5% CO_2_ incubator with the induction media being replenished at a regular interval of 2–3 days [28]. For chondrogenic lineage differentiation analysis, four percent paraformaldehyde was used to fix the cells and it was marked for mucopolysaccharides with the help of 0.1 percent toluidine blue (TB). Four experimental groups were created to assess chondrogenic differentiation: Group 1, control; Group 2, induction media; Group 3, 1 µM of melatonin in induction media; and Group 4, 25 µM of melatonin in induction media.

### 2.9. Real-Time Quantitative Polymerase Chain Reaction (qRT-PCR) for Gene Expression Analysis

The adipogenic (on Day 21), osteogenic (on Day 21), and chondrogenic (on Day 30) capabilities of the cells were subjected to gene expression analyses. A Thermo Scientific GeneJET Ge-nomic Ribonucleic Acid Purification Kit (Lithuania) was employed to extract total RNA. The extracted RNA was reverse-transcribed and a quantitative real-time polymerase chain reaction system (QuantStudio5, CA, USA) with SYBRGreen master mix (Applied Biosystems, Houston, TX, USA) was used to quantify the genes of interest: (1) for adipogenesis: PPARG (peroxisome proliferator activated receptor gamma), LPL (lipoprotein lipase), and CEBPA (CCAT/enhancer binding protein alpha); (2) for osteogenic differentiation: RUNX2 (runt-related transcription factor-2), COL1A1 (collagen 1 A1), ALPL (alkaline phosphatase), OCN (osteocalcin), OPN (osteopontin); and (3) for chondrogenic differentiation: SOX9 (SRY-box transcription factor 9), COL2A1 (collagen 2 A1), ACAN (aggrecan). These genes were normalized to the expression of glyceraldehyde 3-phosphate dehydrogenase (GAPDH) as a housekeeping gene by employing the ΔΔCt method for relative gene expression analysis. The primer-sequence list for the genes of interest (Integrated DNA Technologies, Coralville, IA USA) is presented in Table 1 [28].

### 2.10. Statistically Analyzed Outcomes

The outcomes were described as the average ± standard deviation (SD). The Prism 8 software (GraphPad, San Diego, CA, USA) was employed for analyzing data. For direct comparison, a *t*-test was adopted, while for the comparison of multiple groups, the ANOVA was implemented. As per statistics, *p* < 0.05 was deemed to be significant, *p* < 0.01 was deemed to be remarkably significant, and *p* > 0.05 was deemed to be not significant.

## 3. Results

### 3.1. Expression of Stem Cell Markers on the Isolated and Cultured Dental Pulp Stem Cells

**Figure 1:**
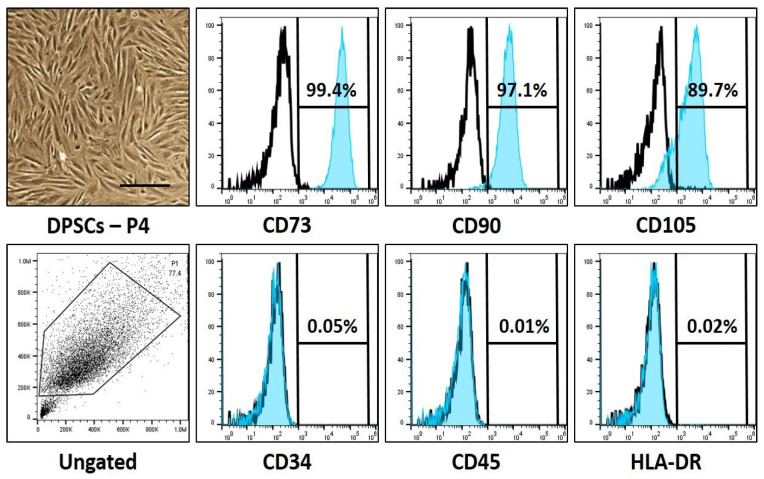
Photomicrographs and characteristic cell surface marker analysis of dental pulp stem cells. Scale bar: 200 μm.

In the present study, we aimed to isolate DPSCs from human third molar teeth, to be considered for flow cytometry analysis to characterize the isolated DPSCs with standard cell surface markers. The flow cytometric sorting of cells disclosed the predominant expression of CD73, CD90, and CD105 (99.4%, 97.1%, and 89.7%, respectively), which are DPSC markers, and poor expression of CD34, CD45, and HLA-DR (0.05%, 0.01%, and 0.02%, respectively) which are designated as hematopoietic stem cell markers (data presented in Table 2 and Figure 1).

### 3.2. Cytotoxic and Proliferative Impact of Melatonin on the Dental Pulp Stem Cells

**Figure 2:**
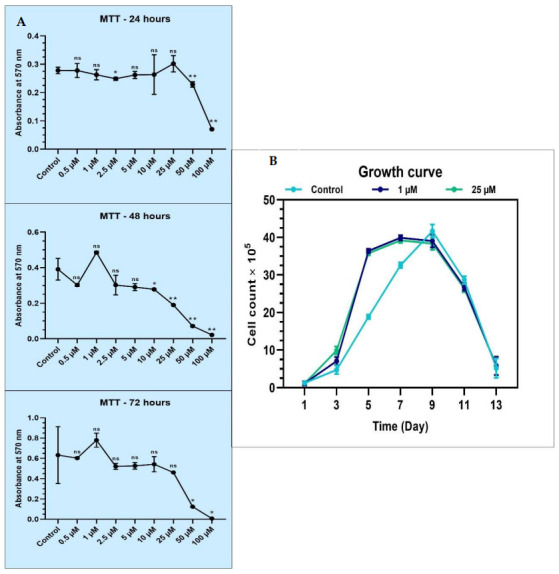
(**A**) MTT assay for comparative cell viability of DPSCs treated with various concentrations of melatonin and comparative population doubling the time of DPSCs treated with low (1 µM) and high (25 µM) concentrations of melatonin. ns, not significant; * *p* < 0.05 and ** *p* < 0.01; (**B**) proliferative ability of DPSCs in the absence (control) and presence of 1 µM and 25 µM melatonin for 13 days.

An MTT assay was performed to assess the cytotoxicity of melatonin on the DPSCs. Based on the MTT assay, ideal concentrations of 1 µM and 25 µM melatonin were chosen for further assays, as these concentrations were well tolerated without inducing toxic effects on the DPSCs (data presented in Table 3 and Figure 2A). It was also found that melatonin did not induce cytotoxic changes on the DPSCs at low (1 µM) as well as high (25 µM) melatonin concentrations.

The proliferative ability of DPSCs in the absence and presence of 1 µM and 25 µM melatonin was studied for 13 days. It was found that at Day 5, there was an increased proliferation rate noted in 1 µM and 25 µM melatonin-treated cells, while at Day 7, a peak was noted in the control untreated cells. The growth curve was almost similar in the 1 µM and 25 µM melatonin treatment groups, revealing that melatonin in these concentrations did not cause significant dose-dependent influence on the proliferative activity of the DPSCs (data in Table 4 and Figure 2B).

### 3.3. Impact of Melatonin on the Adipogenic, Osteogenic, and Chondrogenic Differentiation Capacities of the Dental Pulp Stem Cells

**Figure 3:**
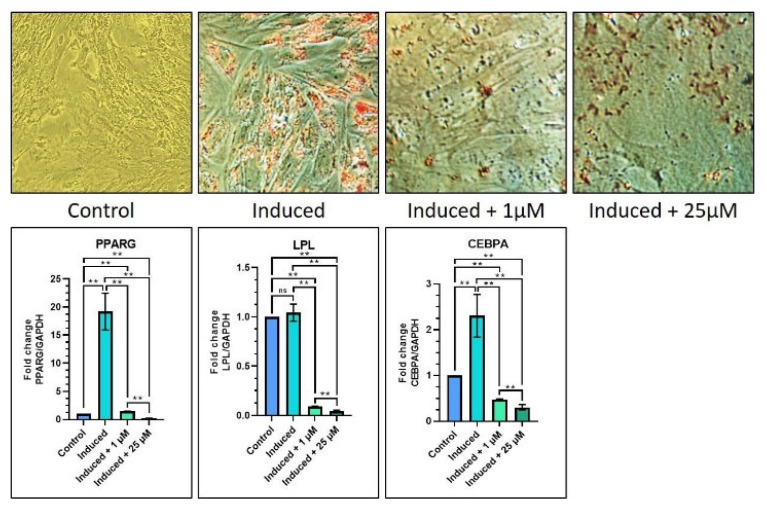
Comparative adipogenic differentiation (oil red O staining) of DPSCs with low (1 µM) and high (25 µM) concentrations of melatonin and RT-qPCR-based gene expression analysis of adipogenesis-related genes. Scale bar: 200 μm. ns, not significant; ** *p* < 0.01.

In order to investigate how melatonin affects the capacity of DPSCs to differentiate into adipocytes, osteocytes, and chondrocytes, a number of previously indicated genes of interest were examined using qRT-PCR. Adipogenic, chondrogenic, and osteogenic differentiation were stimulated using an induction medium containing melatonin at concentrations of 1 and 25 µM. The different experimental groups were: control (Group 1), induction media (Group 2), induction media with 1 µM melatonin (Group 3), and induction media with 25 µM melatonin (Group 4). Considering adipogenic differentiation, it was discovered that PPRAG, LPL, and CEBPA levels were lower in the induction media with 1 M melatonin and the induction media with 25 M melatonin groups as compared with the induction media and the control groups (p 0.05). The results of the staining experiment might support those of the gene expression study (Table 5 and Figure 3).

**Figure 4:**
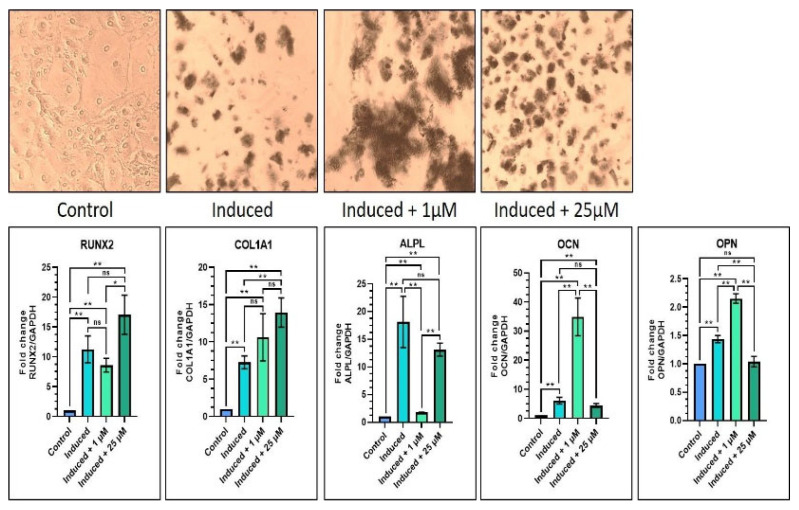
Comparative osteogenic differentiation (von Kossa staining) of DPSCs with low (1 µM) and high (25 µM) concentrations of melatonin and RT-qPCR-based gene expression analysis of osteogenesis-related genes. Scale bar: 200 μm. ns, not significant; * *p* < 0.05 and ** *p* < 0.01.

Regarding osteogenic differentiation, it was found that, in the induction media with 1 µM melatonin, the results showed significant increases in COL1A1, OCN, and OPN gene expressions as compared with the control batch (*p* < 0.05), and significant increases in OCN and OPN gene expressions (*p* < 0.05) as compared with the induction media batch. Similarly, in the induction media with 25 µM melatonin, there were notable increases in all the previously listed genes as compared with the control group. As compared with the induction media group, there was a notable elevation in RUNX2 and COL1A1 gene expressions (*p* < 0.05), and a significant decrease in OPN gene expression (*p* < 0.05). It was also observed that the induction media with the 25 µM melatonin group showed a significant increase in ALPL gene expression (*p* < 0.05) as compared with the induction media with the 1 µM melatonin group, while all other genes were not affected significantly (Table 6 and Figure 4). The von kossa staining revealed increased staining intensity with the 1 µM and 25 µM melatonin groups as compared with Groups 1 and 2 (Figure 4).

**Figure 5:**
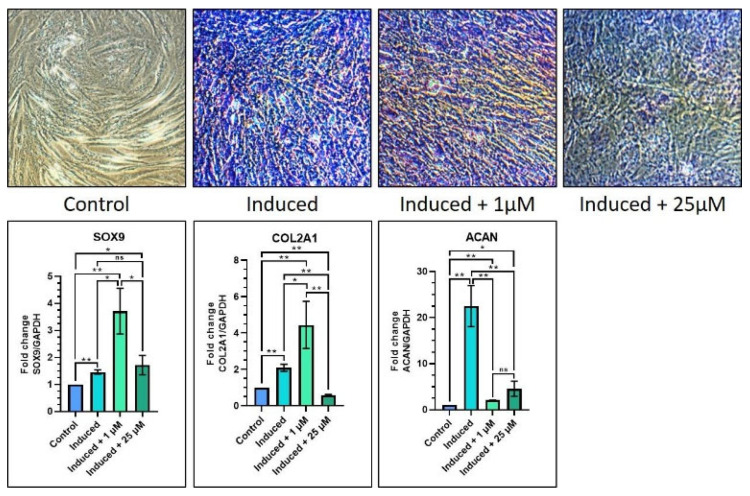
Comparative chondrogenic differentiation (toluidine blue staining) of DPSCs with low (1 µM) and high (25 µM) concentrations of melatonin and RT-qPCR-based gene expression analysis of chondrogenesis-related genes. Scale bar: 200 μm. ns, not significant; * *p* < 0.05 and ** *p* < 0.01.

With regard to chondrogenic differentiation, there was a significant increase in SOX9, COL2A1, and ACAN gene expressions in 1 µM melatonin (Group 3) as compared with the control (Group 1) (*p* < 0.05). When equated to the induction media Group 2, the induction media with 1 µM melatonin Group 3 caused a significant increase in SOX9 and COL2A1 geneexpression (*p* < 0.05), while there was a notable reduction in ACAN gene expression (*p* < 0.05). In addition, the induction media with 1 µM melatonin had significantly increased expression of COL2A1 and ACAN (*p* < 0.05) (Table 7 and Figure 5). The toluidine blue staining images showed a similar trend among Groups 2, 3 and 4, with low staining intensity when compared with the abovementioned groups (Figure 5).

## 4. Discussion

The present research was performed with the aim of investigating the impact of melatonin on the proliferative ability, and the adipogenic, osteogenic, and chondrogenic differentiation capacities of DPSCs. As a part of the investigation process, the dental pulp of healthy human volunteers (*n* = 12) was considered for isolating the stem cells. The isolated DPSCs were cultured and the flow cytometry technique was utilized to characterize them based on cell surface marker expressions. While the cells predominantly expressed CD105, CD73, and CD90 (MSC markers), they failed to express CD45, CD34, and human leukocyte antigen-DR isotype (hematopoietic SC markers). The former findings explain the mesenchymal characteristics of the DPSCs and are in accordance with previously performed studies that have emphasized that DPSCs are mesenchymal stem cells [29,30,31].

Further, we performed an MTT assay using melatonin at various concentrations to assess its cytotoxic effects on the cultured and characterized DPSC. The study findings revealed that melatonin was well tolerated by the cells and did not exert toxic effects at low (1 µM) and high (25 µM) concentrations. These findings are acceptable as melatonin by itself is a cytoprotective agent and protects cells from harmful external stressors [32]. Based on the findings of the MTT assay, 1 µM melatonin and 25 µM melatonin concentrations were used for further assays.

Regarding the assessment of proliferative ability, the DPSCs showed similar proliferative rates under the influence of 1 µM and 25 µM melatonin. While there was a spike on Day 5 in the melatonin treatment groups, there was a spike on Day 7 in the untreated cells. This finding revealed that melatonin had a significant influence on the proliferative activity of DPSCs and was in agreement with a previous study that identified melatonin influenced proliferation of mesenchymal stem cells [33].

In connection with the assays to measure adipogenic, chondrogenic, and osteogenic capabilities of DPSCs under the influence of melatonin, we used untreated control cells (Group 1), induction media (Group 2), induction media with 1 µM melatonin (Group 3), and induction media with 25 µM melatonin (Group 4). The results of the adipogenic differentiation assay revealed that melatonin significantly reduced or inhibited adipogenic differentiation of the DPSCs in the presence of both 1 µM and 25 µM melatonin, as assessed by the reduced expressions of PPRAG, LPL, and CEBPA gene expression. Our findings corroborated with a study that demonstrated reduced expression of adipogenic orchestrating genes in adipogenic SCs under the influence of melatonin and vitamin D [34]. Another study demonstrated that melatonin retarded adipogenic proliferation of human MSCs via dysregulation of RUNX2 and PPAR gamma gene expression [35]. Taking collective evidence from the above studies, it can be concluded that melatonin is an anti-adipogenic factor in stem cell applications.

Likewise, in the osteogenic differentiation assays, it was found that in the induction media with 1 µM melatonin and the induction media with 25 µM melatonin groups, there was a significant increase in all osteogenesis genes as compared with the control. However, when equated to the induction media (Group 2), there was a discordant expression in the genes in both melatonin groups. It was found that in the presence of 1 µM melatonin, there was an increase in RUNX2, OCN, and OPN gene expression and a reduction in ALPL gene expression, while in the 25 µM melatonin, there was an increase in RUNX2, COL1A1 gene expression and a decrease in ALPL, OCN and OPN gene expression. It was also observed that 25 µM melatonin showed a greater increase in ALPL as compared with 1 µM melatonin (Group 3). The finding of the differential effects of melatonin on osteogenic genes is a novel and interesting finding. However, at the phenotypic level, the present study demonstrated intense staining was noticed with the von Kossa dye in both the melatonin groups (Groups 3 and 4). This finding is, again, very significant, as melatonin was found to be a potent osteodifferentiation molecule, as revealed by the gene expression and staining results. The results are justifiable as melatonin could act on other genes of interest in the DPSCs, driving them towards osteogenic lineage. This fact has been demonstrated by a study wherein MSCs differentiated into osteocytes on melatonin, promoting BMP-9 through the beta-catenin signaling pathway [36]. Another study demonstrated that melatonin ameliorated the effect of reactive oxygen species and impaired the MSCs (derived from the bone marrow) to differentiate into osteocytes when TNF alpha was present [37].

In chondrogenic differentiation, there was increased SOX9 and COL2A1 gene expression and decreased ACAN gene expression in the induction media with 1 µM melatonin group as compared with the induction media (Group 2). Another contradicting finding is that the three abovementioned genes were upregulated in the induction media with 1 µM melatonin group and downregulated in the induction media with 25 µM melatonin group. The toluidine blue staining results clearly demonstrated low staining intensity in the 25 µM melatonin (Group 4) as compared with Groups 2 and 3. From the above findings, it could be elucidated that melatonin does not cause dose-dependent effects on chondrogenic differentiation of DPSC. The current research is one of the earliest to assess the chondrogenic differentiation of DPSC under the influence of melatonin.

## 5. Conclusions

Overall, from our investigation, it appears that melatonin can significantly cause osteogenic differentiation of DPSCs through multiple pathways and does not influence adipogenic and chondrogenic differentiation effects. Thereby, melatonin could be used in stem cell and tissue engineering applications as a differentiation promoter, cytoprotector, and antioxidant, especially in the context of bone regeneration where results seem to be promising. Future clinical studies on animal and human models using melatonin-treated stem cells are required to assess the clinical tangibility of the concept and would aid in exploiting the complete benefits of melatonin as a stem cell promoter molecule.

## Figures and Tables

**Table 1:** List of primers.

Gene	Forward Primer	Reverse Primer
PPARG	5′-AGC CTG CGA AAG CCT TTT GGT G-3′	5′-GGC TTC ACA TTC AGC AAA CCT GG-3′
LPL	5′-CTG CTG GCA TTG CAG GAA GTC T-3′	5′-CAT CAG GAG AAA GAC GAC TCG G-3′
CEBPA	5′-AGG AGG ATG AAG CCA AGC AGC T-3′	5′-AGT GCG CGA TCT GGA ACT GCA G-3′
RUNX2	5′-GTG CCT AGG CGC ATT TCA-3′	5′-GCT CTT CTT ACT GAG AGT GGA AGG-3′
COL1A1	5′-GAT TCC CTG GAC CTA AAG GTG C-3′	5′-AGC CTC TCC ATC TTT GCC AGC A-3′
ALPL	5′-GCT GTA AGG ACA TCG CCT ACC A -3′	5′-CCT GGC TTT CTC GTC ACT CTC A-3′
OCN	5′-GGC GCT ACC TGT ATC AAT GG-3′	5′-TCA GCC AAC TCG TCA CAG TC-3′
OPN	5′-CGA GGT GAT AGT GTG GTT TAT GG-3′	5′-GCA CCA TTC AAC TCC TCG CTT TC-3′
SOX9	5′-GCC GAA AGC GGG CTC GAA AC-3′	5′-AAA AGT GGG GGC GCT TGC ACC-3′
COL2A1	5′-CCT CCA GGT CTT CAG GGA AT-3′	5′-AGG AGG TCC AAC TTC TCC CT-3′
ACAN	5′-GCG AGT TGT CAT GGT CTG AA-3′	5′-TTC TTG GAG AAG GGA GTC CA-3′
GAPDH	5′-GTC TCC TCT GAC TTC AAC AGC G-3′	5′-ACC ACC CTG TTG CTG TAG CCA A-3′

**Table 2:** Flow cytometry analysis of MSC specific markers in DPSCs.

MSC Markers	Percentage Positive Cells
CD73	99.4%
CD90	97.1%
CD105	89.7%
CD34	0.05%
CD45	0.01%
HLA-DR	0.02%

**Table 3:** MTT assay.

Melatonin Concentration	MTT 24 h	*p*-Value (vs. Control)	MTT 48 h	*p*-Value (vs. Control)	MTT 72 h	*p*-Value (vs. Control)
Control	0.278 ± 0.012	-	0.391 ± 0.061	-	0.632 ± 0.280	-
0.5 µM	0.278 ± 0.025	0.9876	0.302 ± 0.007	0.065	0.603 ± 0.009	0.8656
1 µM	0.263 ± 0.018	0.2869	0.485 ± 0.007	0.055	0.780 ± 0.068	0.4253
2.5 µM	0.249 ± 0.006	0.0174	0.302 ± 0.055	0.1337	0.521 ± 0.029	0.5325
5 µM	0.262 ± 0.013	0.1808	0.291 ± 0.020	0.0526	0.526 ± 0.032	0.5518
10 µM	0.264 ± 0.070	0.7416	0.278 ± 0.006	0.0322	0.542 ± 0.075	0.6192
25 µM	0.302 ± 0.029	0.2591	0.190 ± 0.003	0.0045	0.461 ± 0.002	0.3498
50 µM	0.229 ± 0.009	0.0047	0.071 ± 0.006	0.0008	0.124 ± 0.003	0.0348
100 µM	0.071 ± 0.003	<0.0001	0.022 ± 0.002	0.0005	0.007 ± 0.001	0.0181

**Table 4:** Growth curve.

Time (Days)	Control	1 μM	25 μM	Control vs. 1 μM	Control vs. 25 μM	1 μM vs. 25 μM
1	1.22 ± 0.56	1.143 ± 0.525	1.123 ± 0.516	No	No	No
3	4.746 ± 1.141	7.067 ± 1.069	9.89 ± 1.051	Sig	Sig	Sig
5	18.855 ± 0.588	36.4 ± 0.551	35.761 ± 0.541	Sig	Sig	No
7	32.577 ± 0.742	39.886 ± 0.695	39.186 ± 0.683	Sig	Sig	No
9	41.657 ± 1.773	39.025 ± 1.661	38.34 ± 1.631	No	No	No
11	28.633 ± 1.022	26.824 ± 0.957	26.353 ± 0.94	Sig	Sig	No
13	5.179 ± 2.592	5.788 ± 2.428	5.687 ± 2.386	No	No	No

No, not signfiicant; Sig, *p* < 0.05.

**Table 5:** Relative gene expression of adipogenesis-related genes.

Gene	PPARG	LPL	CEBPA
Control	1 ± 0	1 ± 0	1 ± 0
Induction media	19.187 ± 3.265	1.044 ± 0.087	2.305 ± 0.466
*p*-Value (vs. control)	0.0006	0.4325	0.0083
Induction media with 1 μM melatonin	0.0010 ± 0.00019	0.00032 ± 0.000063	0.00042 ± 0.000082
*p*-Value (vs. control)	<0.0001	<0.0001	<0.0001
*p*-Value (vs. induction media)	0.0007	<0.0001	0.0024
Induction media with 25 μM melatonin	0.187 ± 0.045	0.044 ± 0.01	0.305 ± 0.058
*p*-Value (vs. control)	<0.0001	<0.0001	<0.0001
*p*-Value (vs. induction media)	0.0005	<0.0001	0.0018
*p*-Value (vs. induction media with 1 μM melatonin)	<0.0001	0.0012	0.0087

**Table 6:** Relative gene expression of osteogenesis-related genes.

Gene	RUNX2	COL1A1	ALPL	OCN	OPN
Control	1 ± 0	1 ± 0	1 ± 0	1 ± 0	1 ± 0
Induction media	11.25 ± 2.266	7.263 ± 0.855	18.12 ± 4.626	6.16 ± 1.127	1.437 ± 0.065
*p*-Value (vs. control)	0.0014	0.0002	0.003	0.0014	0.0003
Induction media with 1 μM melatonin	8.605 ± 1.127	10.62 ± 3.157	1.791 ± 0.065	34.889 ± 6.463	2.152 ± 0.084
*p*-Value (vs. control)	0.0003	0.0062	<0.0001	0.0008	<0.0001
*p*-Value (vs. induction media)	0.1444	0.15	0.0036	0.0016	0.0003
Induction media with 25 μM melatonin	17.053 ± 3.266	13.938 ± 1.956	13.109 ± 1.162	4.455 ± 0.69	1.039 ± 0.092
*p*-Value (vs. control)	0.001	0.0003	<0.0001	0.001	0.504
*p*-Value (vs. induction media)	0.0648	0.0056	0.1427	0.0891	0.0036
*p*-Value (vs. induction media with 1 μM melatonin)	0.0133	0.1966	<0.0001	0.0013	0.0001

**Table 7:** Relative gene expression of chondrogenesis-related genes.

Gene	SOX9	COL2A1	ACAN
Control	1 ± 0	1 ± 0	1 ± 0
Induction media	1.463 ± 0.082	2.09 ± 0.196	22.472 ± 4.467
*p*-Value (vs. control)	0.0006	0.0006	0.0011
Induction media with 1 μM melatonin	3.716 ± 0.847	4.445 ± 1.296	2.075 ± 0.046
*p*-Value (vs. control)	0.0051	0.01	<0.0001
*p*-Value (vs. induction media)	0.0101	0.0358	0.0014
Induction media with 25 μM melatonin	1.715 ± 0.356	0.585 ± 0.047	4.55 ± 1.626
*p*-Value (vs. control)	0.0254	0.0001	0.0194
*p*-Value (vs. induction media)	0.2981	0.0002	0.0028
*p*-Value (vs. induction media with 1 μM melatonin)	0.0196	0.0067	0.0579

## Data Availability

Not applicable.
